# Coping with Daily Thermal Variability: Behavioural Performance of an Ectotherm Model in a Warming World

**DOI:** 10.1371/journal.pone.0106897

**Published:** 2014-09-10

**Authors:** José M. Rojas, Simón B. Castillo, Guillermo Folguera, Sebastián Abades, Francisco Bozinovic

**Affiliations:** 1 Departamento de Ecología and Center of Applied Ecology & Sustainability (CAPES), Facultad de Ciencias Biológicas, P. Universidad Católica de Chile, Santiago, Chile; 2 Centro de Investigación e Innovación para el Cambio Climático, Universidad Santo Tomás, Santiago, Chile; 3 Filosofía de la Biología Group, Facultad de Ciencias Exactas y Naturales & Facultad de Filosofía y Letras, Universidad de Buenos Aires, Buenos Aires, Argentina; 4 Instituto de Ecología y Biodiversidad, Santiago, Chile; The University of Adelaide, Australia

## Abstract

Global climate change poses one of the greatest threats to species persistence. Most analyses of the potential biological impacts have focused on changes in mean temperature, but changes in thermal variance will also impact organisms and populations. We assessed the effects of acclimation to daily variance of temperature on dispersal and exploratory behavior in the terrestrial isopod *Porcellio laevis* in an open field. Acclimation treatments were 24±0, 24±4 and 24±8°C. Because the performance of ectotherms relates nonlinearly to temperature, we predicted that animals acclimated to a higher daily thermal variation should minimize the time exposed in the centre of open field, – i.e. increase the linearity of displacements. Consistent with our prediction, isopods acclimated to a thermally variable environment reduce their exploratory behaviour, hypothetically to minimize their exposure to adverse environmental conditions. This scenario as well as the long latency of animals after releases acclimated to variable environments is consistent with this idea. We suggested that to develop more realistic predictions about the biological impacts of climate change, one must consider the interactions between the mean and variance of environmental temperature on animals' performance.

## Introduction

Current environmental climate variability associated to global change poses one of the greatest threats to organismal functional diversity [1], [2]. Anthropogenic impacts on the earth's climate and habitats will likely increase not only in mean temperature but in the frequency of extremely high temperatures and seasonal/daily variability in certain regions [3]. Although ecologists widely recognize the potential impacts of warming, less attention has been paid to changes in thermal variation on a scale that pertains directly to organisms [4], [5], [6]. Observations and experiments that quantify not only the effect of increases in mean environmental variables but also the effect of environmental variation on organismal traits are important for inferring ecological and evolutionary responses to climate change and the mechanisms by which organisms cope with this variation [7], [8]. In this vein, how behavioural traits are affected by acclimation to temperature variability and their consequences for population persistence is poorly known [9]. Understanding the behavioural responses of animals, and their fitness consequences in variable environments, is important to predict the consequences of global warming on biodiversity. Indeed, as recently suggested by Sih [10] and Wingfield [11], the capability to explain and predict how organism respond behaviourally to human-induced rapid environmental changes has important implication for wildlife management as well as is becoming a focal point of basic and applied research within scenarios of global warming.

Terrestrial isopods are good models to test hypotheses in global change biology, since they exhibit a broad distribution under diverse abiotic conditions [12], [13], [14], [15]. As suggested by Warburg et al. [16] and Hassall et al. [17] the main challenge for terrestrial isopod' fitness is to cope with desiccation stress and temperature fluctuation. In this way, some authors have evidenced the plasticity of diverse traits – behaviour included – that would help the persistence of woodlouse along different geographic as well as -climatic conditions [18], [19], [20], [21], [22], [23], [24]. It is common to observe seasonal changes in its daily activity pattern, together with the use of shelters and the formation of aggregations that hypothetically allows woodlice to avoid unfavourable environmental conditions [16], [25], [26]. Formally, the locomotory activity of terrestrial isopod is described as an uncorrelated discrete Random Walk [27], [28], [29], [30]. This kind of displacement indicate that these animals moves straight ahead for a certain distance, then turn over a random angle (turning angle), and again moves straight ahead to turn without directional persistence [27], [28]. The setting of this type of movement is mainly determined by the degree of autocorrelation of the angles of rotation. An increase in this variable determines a more linear movement of individuals. In laboratory studies, an increment in the autocorrelation level is associated mainly to an escape response, characterized by the temporal persistence of autocorrelation [28], [30], [31], [32], [33]. In the field, the search of suitable patches (e.g. soil moisture) or a dispersion behaviour is also characterized by a significant autocorrelation of turning angle but with a more unpredictable temporal memory, establishing more complex activity patterns such as correlated random walks or spiral displacement[29], [30], [34], [35], [36].

**Figure 1:**
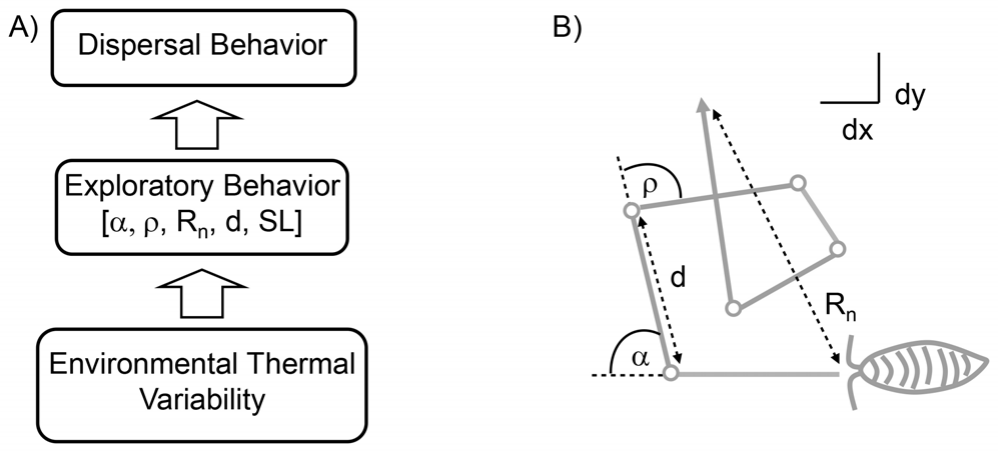
Dependence of behaviour of space use on the environmental thermal variability. (A). Dispersion trajectory is made by steps or SL (B).The minimal length is determined by the interval sampling of each behavioural record, i.e. gray line between open circles. Circles represent a sampling event. The absolute angle (α) is defined between the horizontal plane (dx) and the step. The relative angle (ρ) or turning, is the angle between successive steps. Distance (d), is the total distance during each record. The mean distance (R_n_) is the net distance between the current location and the first relocation of the trajectory. Modified from [63].

Here we experimentally tested the effect of acclimation to environmental thermal variability on dispersal behaviour in the terrestrial isopod *Porcellio laevis* in an open field. Traditionally, the inclusion of the behaviour in studies of thermal acclimation involves two kind of approaches, namely temperature selection [37], [38], [39] and locomotory performance [40]. In all cases, behaviour is used as descriptor of the flexibility or sensitivity of a whole-individual to a new thermal regime, contributing to understand process such as adaptation [37], [41]. Nevertheless, behavioural responses behind ecological relationship of individuals or its implication on fitness still puzzling. Organismal studies in *P. laevis* evidence plasticity in physiological and life history traits in response to different geographic-climatic conditions [19], [20], [21], [23], [24]. Particularly, when the climatic conditions imply thermal variation, a decrease in locomotor and development performance is described in animals acclimated to an increment in the amplitude of ambient temperature variation [7], [8], [40]. Based on these data, and assuming that dispersal behaviour of *P. leavis* fits to a discrete random walk model, going from linear displacements under stressful condition to a more complex at normal condition [27], [28], [32], [34], we predict that animals acclimated to a higher daily thermal variation should minimize the time exposed in the center of open field or in other words to increase the linearity of displacement. Consequently, Fig 1 shows that a dispersion trajectory is made by steps and the minimal length is determined by the interval sampling of each behavioural record, i.e. – the black line between open circles. Circles represent a sampling event. The absolute angle (α) is defined between the horizontal plane (dx) and the step. The relative angle (ρ) or turning, is the angle between successive steps. Distance (d), is the total distance during each record. The mean distance (R_n_) is the net distance between the current location and the first relocation of the trajectory (Fig. 1). In our view this kind of approaches will help to understand if species physiological and behavioral flexibility may buffer (or not and how) the effect of global warming.

## Material and Methods

### Ethics Statement

The study was performed with *P. laevis* specimens collected in the Mediterranean habitats of central Chile at San Carlos de Apoquindo, a Field Station of the Department of Ecology, Catholic University of Chile (33°23′ S, 70°31′ W at 1,230 m above sea level).

Experimental protocols followed the rules of The Ethics and Biosafety Committee of the Faculty of Biological Sciences at the Catholic University of Chile, permit No. CBB-100/2012. *Porcellio leavis* is not an endangered or protected species. We used animals of similar body size and both sexes.

### Animals and experimental design

The mean minimum air temperature at San Carlos de Apoquindo is nearly 6°C below the mean and the maximum goes 7°C higher, on average [42]. Respect to yearly seasonality, the broadest range of temperatures is observed in Summer (16–17°C between mean maximum and mean minimum temperature), and the narrowest in Autumn and Winter (9–10°C).

After collection, animals were sorted by sex and maintained in plastic Petri dish (50 mm diameter; with a base layer of plaster-of-paris to maintain humidity). Food, in the form of dry spinach, and water was provided ad libitum [7]. Pregnant females were identified in the laboratory and placed under standard conditions of light (L∶D = 12∶12) and temperature (24°C) in culture boxes (2.2×2.2×2.4 cm) with a layer of damp sand one cm thick. Previous reports indicated that *P. laevis* exhibit a life cycle of about 12–18 months [43]. Thus, at first 10 days of development, F1 were maintained at same conditions as parents at 24°C to avoid higher rates of mortality [7]. Following Folguera et al. [7] methodology during 74 days, 60 individuals were randomly assigned to one of three thermal treatments in climatic chambers (20 replicates); namely constant temperature regime at 24°C (δ = 0) and two treatments with variance in temperatures, reaching a maximum during daytime and a minimum at night with alternating temperature regimes of 28–20°C (δ = 4) and 32–16°C (δ = 8). These experimental temperatures were chosen because they are within the thermal tolerance range and daily variability of *P. laevis* [20], [21], [22], [44].

Dispersion behaviour of woodlice was evaluated in an open-field setup. The open field consisted in a circular surface of 45 cm radius made of dry plaster and surrounded by a wall of opaque acrylic. Above the open-field we placed a webcam connected to a notebook which allow us to record animals behaviour. Studies were conducted in an acclimated room at 24°C. Displacement of animals was recorded during 15 minutes. Its location (X-Y coordinate) into the open field was determined using a video tracking system (Smart 2.0 – Panlab, Barcelona Spain) at 0.2 intervals seconds (event sampling). Previously to each record, each individual was placed in the centre of field within a dark chamber during 5 minutes to reduce handling stress. Upon the end of these observations, all experimental woodlice subjects were released back at their original sites of capture.

### Movement analysis

To compare dispersal behaviour among treatments, we considered that displacement of woodlice follow basically a discrete random walk, without directional correlation. Therefore, a core point of analysis corresponded to checking randomness in the lineal and angular parameters of each paths (Fig. 1) Thus, each woodlouse was characterized by a trajectory. From the trajectory we estimated the parameters indicated in Fig. 1. Net displacement was quantified by the net square displacement (R^2^_n_) see [29], [45]. Each movement variable included the elapsed time since each animal started the displacement until they reached the wall of the open field. As an additional descriptor of spatial behaviour we used the latency to first displacement (seconds). The exploration level or tortuosity of each path we estimated using the mean fractal dimension (D). This value varied from 1 (straight line) to 2 (a travel path which is so tortuous that it completely covers a two-dimensional plane, see [46], [47]). The length step, R^2^n, and turn angle (relative and absolute) for each path on each interval were estimated using the adehabitat package for R-Cran software [48]. The fractal dimension was estimated with the Fractal Mean estimator using a window range (or frame) of 0.25 to correct the border effect (see [49]) implemented in the Fractal software (Fractal ver. 5, V. Nams, Nova Scotia Agricultural College).

### Statistical analysis

A generalized lineal model was used to test differences in the fractal dimension of displayed by isopods. Thermal treatment was used as categorical variable and body size as weight variable [50], [51]. A similar statistical approach was used to evaluate the effect of acclimation temperature regime on the turning angle, absolute angle, step size and net distance. For each trajectory the temporal sequence of each parameter was evaluated as a time series. The median value of each time series was used as a statistical descriptor the each parameter, assuming that the median follows a gamma distribution with a log function as link [52]. The fractal dimension was also evaluated using a generalized lineal model, but in this case, using the mean as the central tendency descriptor. The significance of models was tested using loglikelihood ratios between the adjusted model and a null model (intercept). The significance of coefficients of models was estimated using an unpaired two tails t-test. Latency and velocity variables were rescaled to log(x). These variables were assessed using a one way ANOVA. Previously the assumptions of each test were evaluated. The temporal autocorrelation level of behavioural descriptors was evaluated using an autocorrelation function analysis (ACF). These analyses were conducted using the autocorrelation descriptors based in the square differences between successive steps to lineal parameters and chord distance between successive angles to test relative angle, estimated by Adehabitat package for the R-Cran software [47]. The significance level of these descriptors was assessed using the 95% confidence intervals estimated from permutation procedure implemented in Adehabitat package. Previously to each test, random and observed values were centred using the average of random values, and then transformed into absolute values [47], [53]. We used correlograms to show mean values by thermal treatment for differences between ACF observed and the lower confidence interval estimated by individual. Finally, the central tendency of the dispersal behaviour was evaluated on frequency distribution of step length and turning angle using the Hartigan's dip test to unimodality implemented in the diptest package for R-Cran [48]. This analysis was pooled all individuals by treatment All parametric tests used were implemented using the R-Cran software (version 2.12.2).

## Results

**Figure 2:**
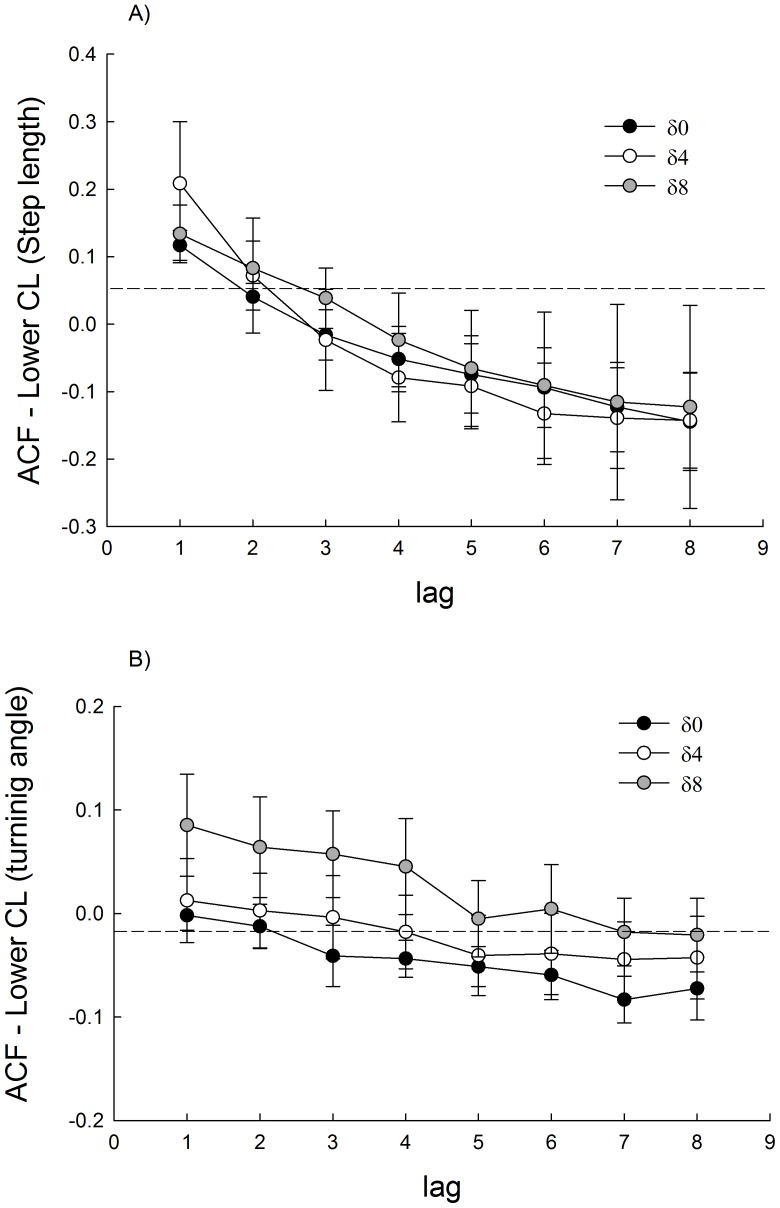
Autocorrelation mean (± standard error) estimated for three thermal treatments of acclimation for step length (A) and relative angle (B). Daily thermal variability treatments are: δ0 = 24±0°C, δ4 = 24±4°C and δ8 = 24±8°C. The ACF were calculated for lag  = 0–8. Segmented line indicated the significance limits. Values correspond only to positive correlations. See text for details.

**Figure 3:**
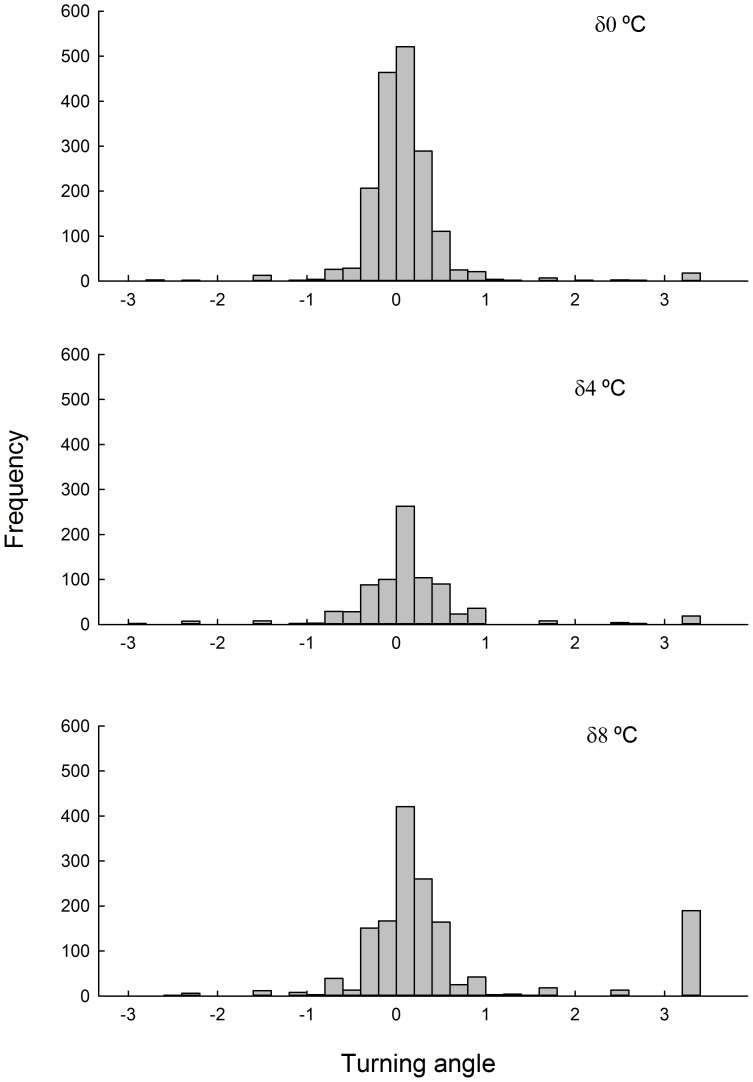
Frequency distribution of turning angle for different thermal treatments. Daily thermal variability treatments are: δ0 = 24±0°C, δ4 = 24±4°C and δ8 = 24±8°C. The Hartigan's dip statistical test revealed a significant bimodality pattern among all treatments. Namely, δ0: D = 0.0214, *P*<0.05; δ4: D = 0.0345, *P*<0.05 and δ8: D = 0.0396, *P*<0.05.

Experimental treatments did not affect body size of individuals (Table 1). Differences in dispersion behaviour as a result of acclimation to different thermal regimes were observed. Animals that experienced the largest thermal variation (δ8) showed a positive autocorrelation in the steps length to a delay of 3 lag. Similarly, the turning angle of these animals showed a lag autocorrelation to 4 (Fig. 2). Animals acclimated to δ0 and δ4 showed positive autocorrelation for the length of the steps to lag 3 only (Fig.2). No autocorrelation on turning behaviour was recorded (Fig. 2). These results were consistent with those observed in the frequency distribution of the relative angles. Animals exposed to a δ0 and δ4 thermal regime showed a symmetrical unimodal distribution centred at 0°. A different pattern is observed in animals acclimated to the larger thermal variation (δ8), that while it is centred at 0°, its distribution is nearly bimodal (Fig. 3).

**Table 1:** Results for one-way analysis of variance for testing the homogeneity in individual body attributes and the general dispersal performance of woodlice among treatments.

	δ0	δ4	δ8	Statistics
Body length (mm)	10.9 (0.3)^a^	11.5 (0.3)^a^	10.8 (0.3)^a^	*F*_(2,57)_ = 1.6; SS = 5.12
Body mass (mg)	71.7 (3.2)^a^	78.4 (3.2)^a^	69.8 (3.2)^a^	*F*_(2,57)_ = 1.9; SS = 820.9
Speed (cm/s)	3.6 (0.4)^a^	5.0 (0.6)^a^	3.8 (0.4)^a^	*F*_(2,57)_ = 1.0; SS = 0.9
Latency (s)	81.1 (16.3)^a^	163.2 (25.4)^b^	100.8 (20.4)^ab^	*F*_(2,57)_ = 4.5; SS = 7.4

Different letters indicate significant differences among treatments (Tukey test at *P*<0.05). Values shown the mean estimated in each thermal variability treatments: δ0, δ4 and δ8 (i.e. 24±0, 24±4 and 24±8°C) for each parameter. In parentheses ±1 standard error.

**Figure 4:**
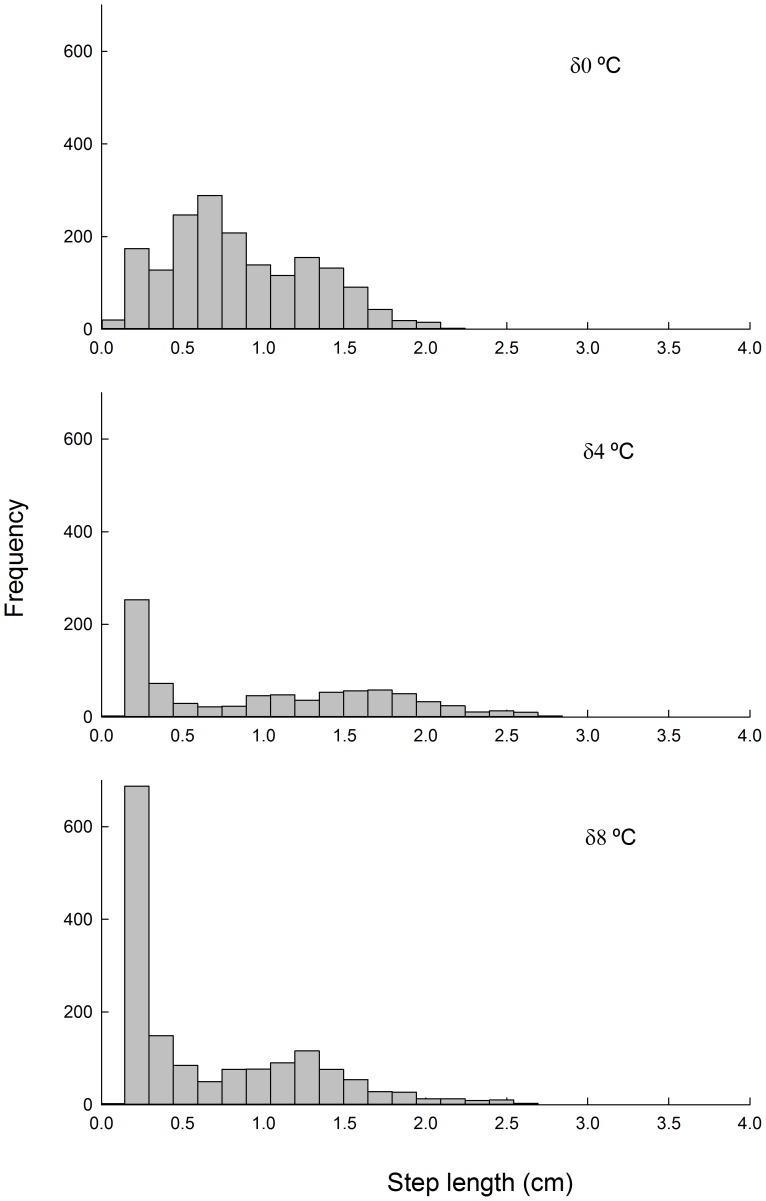
Frequency distribution of step length for different thermal acclimation conditions. Daily thermal variability treatments are: δ0 = 24±0°C, δ4 = 24±4°C and δ8 = 24±8°C. The Hartigan's dip statistical test revealed a significant bimodality pattern among all treatments. Namely, δ0: D = 0.013, *P*>0.05; δ4: D = 0.0123, *P*>0.05 and δ8: D = 0.0614, *P*<0.05.

In the case of the frequency distribution of the length of steps, a tendency to a bimodal distribution was observed, but mainly in animals acclimated to a daily variable temperature (Fig. 4). Particularly, isopods acclimated to highly variable environments (δ8) showed a distribution characterized by a predominance of short steps (Fig. 4). Animals acclimated to δ4 exhibited similar results described for δ8, but with a smaller representation of short steps. Finally, animals acclimated to a stable environment showed a more homogeneous distribution of length steps

**Figure 5:**
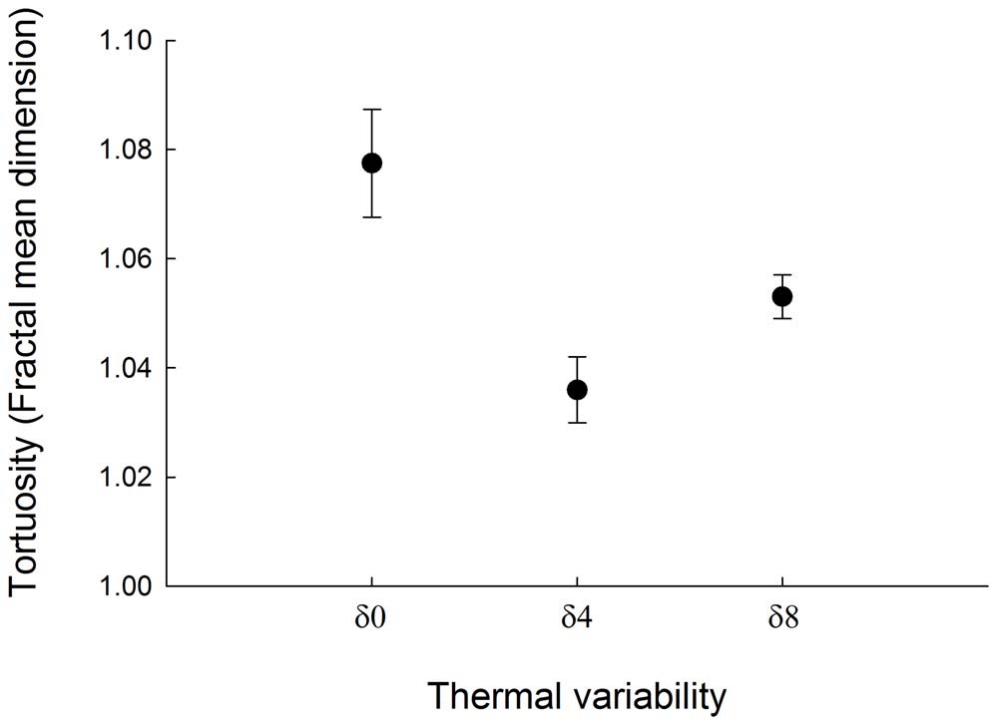
Fractal mean dimension recorded for animals acclimated to different variability thermal regimen. Daily thermal variability treatments are: δ0 = 24±0°C, δ4 = 24±4°C and δ8 = 24±8°C. Bar showed ±1 standard error.

A significant adjust of the generalized linear models was only detected for the descriptors of fractal dimension and absolute angle (Table 2). Thus, no differences were observed in the relative angle (turning) of paths among experimental groups (Table 2). Likewise, no significant differences in step length and net distance travelled (R^2^n) were detected among experimental conditions (Table 2). Regarding to absolute angles, animals exposed to a constant thermal regime showed a significantly lower absolute angle in comparison to animals facing δ4 regimes (Tables 2, 3). Animals acclimated to a δ8 thermal regime did not change the magnitude of absolute angle (Tables 2, 3). A congruent for the absolute angle was observed for fractal dimension. That is, animals that did not experienced thermal variation showed significant greater displacement (i.e. fractality) in the open field compared with animals that experienced a thermal variability of medium intensity (δ4) (Tables 2, 3, Fig. 5). No differences in the degree of fractality of movement between animals that were exposed to δ4 and δ8 were observed (Tables 2, 3, Fig. 5). When assessing the performance of animals, isopods acclimated to an environment without variability showed a latency to first displacement significantly lower than that observed in animals exposed to the δ4 treatment and similar to those acclimated to δ8 conditions (Table 1). Animals acclimated at δ4 and δ8 exhibited no difference in the latency time (Table 1). Finally, no statistical differences were recorded in speed among animals acclimated to different thermal regimes (Table 1).

**Table 2:** Results of generalized linear model for displacement descriptors.

Movement Descriptors	Null Model		Adjusted Model			*P*-value	AIC
	df	Loglik	Df	Loglik	L ratio		
Fractal dimension	5	49.3	7	7.9	15.91	0.001	−89.9
Absolute angle	5	−83.4	7	−79.5	7.92	0.02	172.9
Turning angle	5	48.1	7	48.7	1.16	0.55	−83.3
Step length	5	−51.9	7	−50.7	2.60	0.27	115.4
Net distance (R^2^n)	5	−314.7	7	−.313.5	2.34	0.31	641.0

AIC corresponds to the Akaike information criterion; df: degree of freedom; Loglik: Loglikelihood estimator; L ratio: loglikelihood ratio Chi-square.

**Table 3:** Coefficients for significant models from Table 2.

Movement Descriptors		Thermal treatments		
		δ0	δ4	δ8
Fractal dimension	Estimate	1.07 (0.01)	−0.03 (0.01)	−0.02 (0.05)
	*t*-test; *P*-value	127.7; 0.001	−2,84; 0.006	−1.97; 0.05
Absolute angle	Estimate	−0.32 (0.15)	0.86 (0.30)	0.39 (0.26)
	*t*-test; *P*-value	−2.10; 0.04	2.79; 0.001	1.46; 0.14

In parentheses standard error of coefficient. Student t-test and *P*-values correspond to hypotheses test of the estimated coefficients (df = 57, 4).

## Discussion

To predict responses to climate change, physiological and behavioural ecologists must understand the patterns of thermal variation and the mechanisms by which animals cope with this variation [6], [10], [11], [54], [55]. As pointed out before, impacts on the earth's climate will likely increase the frequency of extremely high temperatures in certain regions [56]. Many organisms are expected to suffer a decrement in performance and fitness [57], [58], but some may preserve their performance through behavioural responses [59]. For instance, we concur with Kearney et al [59] who enlighten the importance of behavioural traits to buffer the impact of global warming, arguing that this is an important and missing element from models of climatic change and predictions of impacts on biodiversity (see also [55]). Accordingly our results with terrestrial isopods represent an example of how changes in behavior allow animals to cope with variable habitats but also how environmental thermal variability may affect behavior.

Helmuth *et al*. [60] analyzed environmental variability at scales relevant to organisms to predict the responses of individuals and populations to thermal variability in the intertidal, where as previously suggested [61] some behavioral traits act as critical phase transition triggered by ambient temperatures. For instance, in hypervariable environments as the intertidal [60], [62], the gastropod *Nerita atramentosa* behaviorally avoid the high temperatures through selection of thermal refuges as well as through huddling. Thus, as terrestrial isopods (this study), the behavior of marine gastropod during emersion seems to be related by local thermal stability or variability of the environment [63]. Theoretically, in variable habitats it is beneficial for ectotherms to select temperatures below thermal physiological optimum [64]. This hypothesis was tested by [65] in the intertidal snail *Chlorostoma funebralis* throughout experimental studies of behavioral thermoregulation in a thermal gradient. They found a “cold-biased” behavior which seems to guide snails to refuges in crevices. Indeed this snail performed a biased random walk along the gradient and huddle together at temperatures near their lower thermal limits. [66], [67] also demonstrated that operative temperature in the intertidal may be one of the leading determinants of time/space variation of behavioral traits in the periwinkle *Echinolittorina peruviana*.

The constraints to behaviour of animals – though changes in ambient temperatures - are probably extremely important in determining when and how a habitat should be used. Climatic effects are of paramount importance since predicted increases in global thermal mean as well as in variance will change many behavioural displays including habitat use and time, among others. Therefore, thermal tolerance should depend on the variance of temperature as well as the mean. Consistent with this view, we found that woodlice exposed to highly variable thermal conditions showed a more conservative displacement. The long correlations of both the turning behaviour as well as the length of steps were greater than those observed in animals exposed to less variable or constant thermal conditions. Particularly for length step, which was not observed in the other test groups were noted. Thus, animals acclimated to a highly variable thermal environment display a more systematic or persistent displacement behaviour when exposed to a new environment, adjusting their behaviour to a more dispersive behaviour, namely less random and more linear [68]. Accordingly, the frequency distributions observed for the linear and angular parameters are consistent with a reduction of the random component in the movement. Among the parameters used to describe the movement of isopods, we consider the use of the fractal dimension, which allows describing the degree of homogeneity in which space was used or explored by the animals during their displacement [49]. Animals acclimated to stable thermal conditions showed a higher degree of scanning in comparison to the other experimental groups. The estimated magnitude for this group is similar to that reported for other terrestrial invertebrates [69], implying that the behaviour of isopods during our study resembles behaviour under natural conditions. This result is consistent with our previous suggestion that animals acclimated to variable conditions exhibit less exploratory behaviour. In this scenario interestingly, animals acclimated to an intermediate thermal variation are situated in an intermediate position. These animals showed a degree of fractalitiy, and bimodality in the frequency distribution of step length, as observed in animals acclimated to highly variable environments. Nevertheless, they also showed a temporal autocorrelation pattern for linear and angular parameters similar to those estimated for animals acclimated to stable environments.

Our results supported that thermal acclimation has significant effects on the behaviour of isopods, a finding not previously reported. Isopods exposed to a thermally variable environment reduce their exploratory behaviour, probably as a way to minimize their exposure to adverse environmental conditions [63], [65]. This scenario as well as the long latency of animals acclimated to variable environments is consistent with this proposal. Clearly, further studies are needed to determine some of the ecological/evolutionary consequences of these behavioural and physiological adjustments, yet our results highlight to the importance of the behavioural component when making predictions about population viability under a climate change scenario. Overall, changes in animals phenologies that result from global warming may produce changes in activity patterns, migration, and breeding timing, and ultimately all of these altered synchronization between trophic levels as well as changed species competitive ability [70]. The emerging picture is that some specific behaviours represent an integrated response to the abiotic environment and its variation and the behavioural ability of animals to respond to new climatic scenarios. According to Sih [10], the observed variation in the behavioural responses in our studied animals may be the result of a cue-response system or because unpredictable environmental information may affect woodlice responses to novel situations or because just behaviour plasticity may affect an animal's response. Probably a signal detection or a cost-benefit hypotheses on variation in learning may be the causes behind our results [5].

A main principle of physiological and behavioural ecology is that populations exposed to environmental change may crash when most individuals deteriorate, and that individuals decline when they reach a state that prevents them from maintaining homeostasis and behaviour –that is, the proper equilibrium through time with internal processes and foreign stimuli. It follows that the effects of climate change on species cannot be assessed from the type, magnitude or time scale of the perturbation, but from the physiological and behavioural states caused by it. Thus, a same set of environmental conditions may be deleterious for one species or population but harmless to another. The relatively narrow approaches used to the study of climate change on biodiversity are limited because they ignore the mechanisms that organisms use to cope with environmental changes [10], [55].
